# New evidence of mating swarms of the malaria vector, *Anopheles arabiensis* in Tanzania

**DOI:** 10.12688/wellcomeopenres.12458.1

**Published:** 2017-09-22

**Authors:** Emmanuel W. Kaindoa, Halfan S. Ngowo, Alex Limwagu, Gustav Mkandawile, Japhet Kihonda, John Paliga Masalu, Hamis Bwanary, Abdoulaye Diabate, Fredros O. Okumu

**Affiliations:** 1 Environmental Health and Ecological Science Department, Ifakara Health Institute, Ifakara, P. O. Box 53, Tanzania; 2 School of Public Health, Faculty of Health Sciences, University of the Witwatersrand, Johannesburg, South Africa; 3 Institut de Recherche en Sciences de la Santé (IRSS)/Centre Muraz, Bobo-Dioulasso 01 BP 545, Burkina Faso; 4 Institute of Biodiversity, Animal Health and Comparative Medicine, University of Glasgow, Glasgow, G12 8QQ, UK

**Keywords:** Anopheles swarms, mating behavior of mosquitoes, malaria, vector control

## Abstract

**Background**: Malaria mosquitoes form mating swarms around sunset, often at the same locations for months or years. Unfortunately, studies of *Anopheles* swarms are rare in East Africa, the last recorded field observations in Tanzania having been in 1983.

**Methods**: Mosquito swarms were surveyed by trained volunteers between August-2016 and June-2017 in Ulanga district, Tanzania. Identified *Anopheles* swarms were sampled using sweep nets, and collected mosquitoes killed by refrigeration then identified by sex and taxa. Sub-samples were further identified by PCR, and spermatheca of females examined for mating status. Mosquito ages were estimated by observing female ovarian tracheoles and rotation of male genitalia. GPS locations, types of swarm markers, start/end times of swarming, heights above ground, mosquito counts/swarm, and copulation events were recorded.

**Results**: A total of 216 *Anopheles* swarms were identified, characterized and mapped, from which 7,142 *Anopheles gambiae *s.l and 13 *Anopheles funestus* were sampled. The *An. gambiae *s.l were 99.6% males and 0.4% females, while the *An. funestus *were all males. Of all *An. gambiae* s.l analyzed by PCR, 86.7% were *An. arabiensis*, while 13.3% returned non-amplified DNA. Mean height (±SD) of swarms was 2.74±0.64m, and median duration was 20 (IQR; 15-25) minutes. Confirmed swarm markers included rice fields (25.5%), burned grounds (17.2%), banana trees (13%), brick piles (8.8%), garbage heaps (7.9%) and ant-hills (7.4%). Visual estimates of swarm sizes by the volunteers was strongly correlated to actual sizes by sweep nets (R=0.94; P=<0.001). All females examined were nulliparous and 95.6% [N=6787] of males had rotated genitalia, indicating sexual maturity.

**Conclusions**: This is the first report of *Anopheles* swarms in Tanzania in more than three decades. The study demonstrates that the swarms can be identified and characterized by trained community-based volunteers, and highlights potential new interventions, for example targeted aerosol spraying of the swarms to improve malaria control.

## Background

Recent successes in malaria control have been mostly attributed to vector control measures, in particular, long-lasting insecticide-treated nets (LLINs) and indoor residual spraying (IRS) ^1^. These tools have contributed to ~78% of all gains achieved since 2000 ^1, 2^. However, effectiveness of these interventions is compromised, partly due to spread of insecticide resistance ^3^ and increased outdoor exposure to mosquito bites among other challenges ^4– 6^. Consequently, persistent malaria transmission still occurs in many places, even where high and sustained coverage of LLINs has been achieved.

To develop new options for malaria vector control, it is important to re-examine the overall ecology of the malaria vectors ^7^, including not only the blood-feeding and resting habits commonly targeted by LLINs and IRS, but also other mosquito habits indoors and outdoors. Mating behavior is one of the most important aspects in the maintenance of species ^8^, yet it is a widely under-investigated aspect of the mosquito biology. Improved understanding mosquito mating systems could possibly provide new opportunities for expanding vector control options. Most insects mate in swarms, whereby dispersed populations aggregate at specific times and places ^9, 10^. In mosquitoes, including the malaria vectors, swarming flights are influenced by presence of visual markers on the ground ^11, 12^. Targeting swarms to deplete mosquito densities indeed offer unrivalled opportunities to drastically reduce mosquito-borne pathogen transmission ^13^. Such an approach has proven effective against some *Anopheles* mosquitoes on a limited scale in Burkina Faso ^14^, but needs to be validated for other vector species in other areas. Besides, mosquito swarms are known to occur perpetually in the same locations at approximately the same time each day ^10, 15^, thus targeting them could be easily achieved by trained community volunteers. The concentrations of males in swarms, predictability and accessibility of the swarming sites and the fact that swarms can be artificially manipulated ^16^, makes the male mosquitoes vulnerable and an easy control target.

While swarms have been commonly identified in west and central Africa ^17– 19^, observations have rarely been reported in East and Southern Africa, most probably because mosquito swarming has not been as thoroughly investigated in this area, as it has been in West Africa. Prior to this current report, there was only a handful of reports, including the seminal work by R.P. Marchand in Tanzania in the early 1980s ^20^, the work by J.D. Charlwood in Mozambique in the early 2000s ^21^, and unpublished observations of *An. arabiensis* swarms in the Kilombero valley in south eastern Tanzania (Dr. Kija Nghabi and Japhet Kihonda, Personal Communication).

This current study was designed to explore the *Anopheles* mating systems in rural south-eastern Tanzania, by assessing occurrence of natural swarming and outdoor nocturnal behaviors of *Anopheles* mosquitoes. The assessment involved accurate identification, mapping and characterizing of mosquito swarms in three study villages in Ulanga district, Tanzania. We assumed that despite lack of previous records, *Anopheles* swarms do indeed occur in the area, and that they can be readily identified and characterized. We used a combination of crowd-sourced community knowledge ^22^, intensive field surveys and expert advice. Our initial objective was therefore to demonstrate natural occurrence of swarms of malaria vectors in rural, south eastern Tanzania and characterize these swarms.

## Methods

### Study area

**Figure 1.**
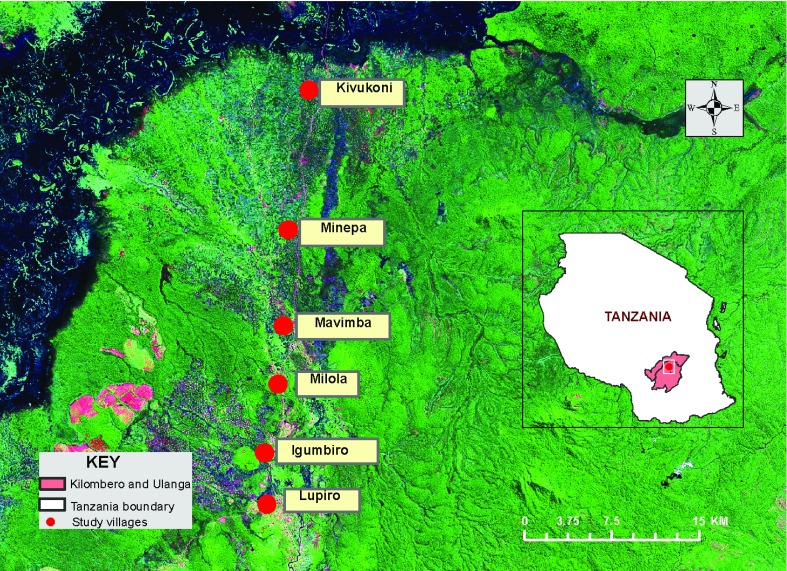
Map of the study area, showing villages in south-eastern Tanzania, where swarm surveys were conducted. Image description: Pan-sharpened mosaic, acquired by Enhanced Thematic Mapper Plus (ETM+) sensor on Landsat 7 Satellite (Courtesy of Donall Cross, University of Aberystwith, UK)).

The study was conducted in rural Ulanga and Kilombero districts, in south eastern Tanzania. Initially, the surveys covered multiple villages in the two districts, but we eventually focused on just three villages of Kivukoni (8.2135°S, 36.6879°E), Minepa (8.2710°S, 36.6771°E) and Mavimba (8.3124°S, 36.6771°E) in Ulanga district, on the Kilombero river floodplain ( Figure 1). The main malaria vectors in the area include *Anopheles funestus* and *An. arabiensis* with minor contributions from *An. rivulorum* ^23^. Overall EIR was last estimated at 4.2 and 11.7 infectious bites/person/year by *An. arabiensis* and *An. funestus* respectively ^23^. The main vector control approach used in the area is LLINs, and the villages benefited from universal LLIN coverage campaigns both in 2011 and in 2016. The main malaria vectors are resistant to pyrethroids used in the LLINs, but still susceptible to organophosphates ^23, 24^. Mean household size is 4.2 ^25^. Most of the houses are mainly mud and brick walled, with thatched or iron-sheet roofs. The communities rely mainly on substance farming, cultivating rice and maize but also fishing.

### Trainings on swarm surveys and characterization

Ten participants were selected to support the project in the three study villages, with the help of community leaders. All participants were male adults aged 18 to 35 years, who had provided written informed consent prior to being enrolled in the study. Initially, the project leaders and research assistants were trained on how to identify mosquito swarms and swarm markers by experienced entomologists from Burkina Faso. The first brief training was conducted in Ifakara, Tanzania, in December 2014, at which stage this project had just been conceived. The second training was done for project leaders during a visit to Burkina Faso in September 2016. All participants in this study, including volunteers searching the swarms, provided written informed consent prior to being enrolled.

**Figure 2.**
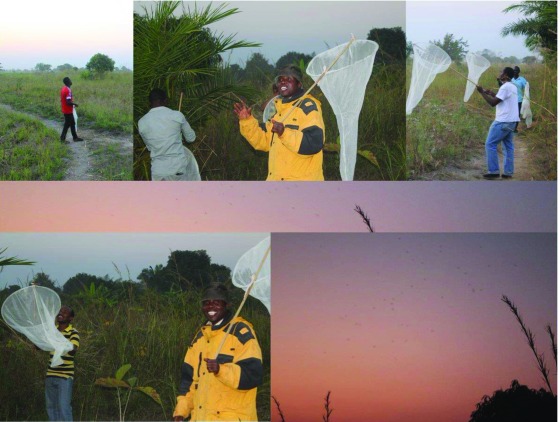
Pictures of volunteers and researchers learning how to identify and sample mosquito swarms in one of the study villages. All participants in the picture provided consent to being photographed.

In April 2016, five months before the second training of project leaders, exploratory swarm surveys were initiated, as an extension of ongoing entomological studies in the study area. During and after these exploratory surveys, the project leaders trained the local community-based swarm searching volunteers on how swarms are identified and characterized, focusing on major features of both the swarms and their associated markers ( Figure 2). This training was conducted over several weeks, and included: i) swarm searching techniques; ii) swarm sampling techniques; iii) swarm characterization and iv) various entomological procedures essential for examining male and female *Anopheles* mosquitoes collected from swarms. All trainings for local community volunteers were conducted in Kiswahili language, which is the common language in Tanzania, and in this specific study area. These volunteers were then relied upon to identify swarms, which the researchers would then follow up for species confirmation.

Further advanced training on swarm surveys and characterization was conducted in Tanzania by a team of experts from Institut de Recherche en Sciences de la Santé (IRSS), Burkina Faso. This advanced training covered: i) swarm searching techniques in dry and wet seasons; ii) swarm visualization techniques; iii) GIS-based analysis of swarms iv) swarm markers and associated environmental features; v) swarm sampling techniques vi) photographic survey of swarms, vii) swarm characterization (including key features to consider); and viii) identification of mating couples in swarms and examination of male and female mosquitoes to identify mated and non-mated individuals.

### Initial exploratory surveys of mosquito swarms

To aid logistical assignment of the swarm searching activities, volunteers were assigned one sub-village to search and report the occurrence of swarms. The trained community volunteers were dispatched to search and report any types of insect swarms in their areas, and report these to the researchers. Every time a swarm was reported by the volunteers, the researchers visited the site immediately (if nearby) or the next day (if in far locations): i) first, to verify whether the swarms were actually present, ii) second, to determine whether they were swarms of mosquitoes as opposed to other insects, and iii) third, to assess whether they were actually *Anopheles* swarms. The first confirmed *Anopheles* mosquito swarm was identified by a community-based swarm searcher in mid-August 2016 in Minepa Village. This provided our initial verified evidence of presence of *Anopheles* swarms in this area. As a result of this initial discovery, the team was reorganized for more intensive swarm searching expeditions. From this point forward, the key objectives now included demonstrating that local community members could be trained to readily identify and characterize *Anopheles* swarms and conduct comprehensive mapping and characterization of *Anopheles* swarms in selected villages. Subsequent efforts were restricted to just three villages (Mavimba, Kivukoni and Minepa) in Ulanga district ( Figure 1), to enable detailed swarm studies.

### Detailed mapping and characterization of *Anopheles* mosquito swarms and swarm markers in three selected villages

After the initial swarm searching, a comprehensive mapping and characterization of the *Anopheles* swarms was conducted in three selected villages, i.e. Minepa, Mavimba and Kivukoni. We worked with volunteers from the different sub-villages, who had been trained on how to spot mosquito swarms and swarm markers, time of day when swarms typically occur, and how to collect mosquitoes using sweep nets to identify the swarming mosquitoes. We relied on lessons learned during the initial exploratory surveys, and progressively optimized our swarm survey techniques. The training provided by experts from Burkina Faso significantly improved the swarm characterization capabilities of the Tanzania team.

Volunteers were assigned specific area (sub-villages) to search for mosquito swarms, by first identifying potential swarm markers in these areas during the day, and then actively searching of mosquito swarms across the area every evening from 18:00Hrs, with the aim of identifying swarms. During the swarm surveys, the volunteers continued to receive supportive supervision and training on how to identify, locate and sample mosquitoes using sweep nets. This continuous training was important since this is the first time *Anopheles* swarms were being recorded in this area. To facilitate the swarm surveys, we also identified, characterized and recorded all potential swarm markers in the study villages. The swarm markers were classified based on the criteria developed by Diabate *et al.,* ^26^, into four categories as follows: a) flat-contrast markers, such as bare land and footpaths, b) flat no-contrast markers, such as grasses and rice fields, c) elevated contrast markers, such as anthill, woodpiles, bricks and garbage bin, and d) elevated no-contrast markers such as banana tree. Then these markers were subsequently monitored in the evenings to identify and record those with swarms.

Following the initial identification and mapping, each verified swarm was comprehensively characterized by the expert team, by repeatedly visiting and examining the swarms at least three times in different days. In addition to recording date, month, and name of village, we followed a systematic process and recorded various pre-determined characteristics as follows: a) heights at which the swarms occurred, (measured as a distance between the base of the swarm and the ground level), b) time of day when the swarming started appearing, recorded to the nearest minute, c) time of night when the swarms were completely dispersed, also measured to the nearest minute; d) physiological status of a sample of mosquitoes collected in the swarm, e.g. mating status and parity, e) any other mosquito species and insects occurring in the same swarm, f) exact geo-location of the swarm marker; measured using handheld GPS receiver (Magellan eXplorist GC, USA), g) ratio of males to females collected in the sample; h) number of copulation events observed in the swarm; i) number and proportion of females caught that have evidence of having been inseminated, j) type of swarm marker; k) parity status of female mosquitoes collected in the swarms, including number and proportion of females that were parous or nulliparous; l) morphological and molecular identification of the species of *Anopheles* mosquitoes collected in the swarm; m) any other unique observations made at the swarm site or on the swarms; and n) approximate number of mosquitoes collected in each swarm. Swarm sizes were estimated in two ways: by sampling with a large sweep net (190 cm diameter), approximately 10 minutes after the start of the swarm, and visually, by the trained volunteers counting the flying mosquitoes and approximating the counts. Lastly, we observed other features which may affect the occurrence of mosquito swarms, time of sunset such as human movements and other insects.

### Laboratory processing of mosquitoes collected in the swarms

Collected mosquitoes (dead or alive) were aspirated from the sweep nets, kept in separate paper cups and maintained on 10% glucose solution so that those still alive could survive till the next morning. The following morning, all mosquitoes were killed in a closed container by freezing, then identified morphologically by taxa and sex, following keys developed by Gillies and Coetzee ^27^. A sub-sample from each collection was further identified by multiplex polymerase chain reaction (PCR) to identify sibling species that were morphologically indistinguishable. The PCR assays were conducted using protocols developed by Scott *et al*., ^28^.

Physiological age of the wild-caught female malaria vectors was approximated based on the status of their ovaries, i.e. whether they had previously laid eggs or not, by observing the coiling or uncoiling of the ovariole tracheoles ^29^. Each unfed female mosquito was first anesthetized in a refrigerator. A drop of distilled water was added to a slide and each specimen kept still on the slide, then the seventh and eighth abdominal segment was pulled using fine needles under stereo dissecting microscope. The ovarial tracheoles were then observed under a compound microscope at 10X objective lens magnification, to determine whether the mosquitoes were parous (tracheoles uncoiled and stretched) or nulliparous (with tracheolar skeins, i.e. coiled) ^29^. 

### Assessment of female insemination status and sexual maturity in males:

To determine the insemination rate, female *An. arabiensis* were dissected under a dissecting microscope and their spermathecae examined for the presence of spermatozoon under a compound microscope using 10X objective lenses ^30, 31^. To assess whether males had matured sexually, genitalia of *An. arabiensis* males were also observed for signs of rotation ^32^.

### Data analysis

Data analysis was done by using R software version 3.0 ^33^. Number of mosquitoes per swarm, duration of swarms, proportion inseminated, proportion parous (or nulliparous) or proportion of males with rotated genitalia were modeled as a function of different variables as follows: village, swarm marker type, month, height of swarms, time of swarming, and volunteer identification. Geolocations of the swarms relative to households and other landmarks were visualized using the ArcGIS 10.0 (ESRI, USA). Percentages of parity status, insemination rate, mean heights of swarms above ground, median duration of swarms, and time of day when swarms started or ended at the different months of surveys, were also calculated. Swarm markers were classified following the classification category developed by Diabate *et al*., ^26^. Visualized volunteer estimates of swarm sizes, and sweep net swarm size estimates were compared and their linear correlation coefficients estimated.

### Ethics statement

Meetings with local leaders were held in the study areas and the main aims of the study were explained by the research team. Written and signed informed consent were obtained from all volunteers participating in the study. All information was given in Kiswahili, the local language. Ethical approval for the study was obtained from Ifakara Health Institute Institutional Review Board (IHI/IRB/No: 38-2016), and from the Medical Research Coordinating Committee (MRCC) at the National Institutes of Medical Research (NIMR), Ref: NIMR/HQ/R.8a/Vol.IX/2428. Approval was also obtained from the Human Research Ethics Committee (Medical) clearance at the University of the Witwatersrand, where EK is registered for PhD (Ethics approval certificate No: M160806). Approval for publishing the manuscript was obtained from the National Institutes of Medical Research (NIMR), Ref: NIMR/HQ/P.12VOL.XXII/24. Printed copies and web links to the publication will be provided to NIMR after publication.

## Results

### Results of exploratory surveys: initial evidence of *Anopheles* swarms

The initial exploration, conducted in multiple villages, revealed that *Anopheles* swarms do indeed occur in villages along the Kilombero river valley in rural south-eastern Tanzania. The surveys also revealed that community members can be relied upon to identify these swarms and that by combining their local knowledge with expert knowledge, the *Anopheles* swarm surveys could be effectively characterized. The first *Anopheles arabiensis* swarm was observed on 15 ^th^ August 2016, in the village of Minepa, by one of the local volunteers. To the best of our knowledge, this is the first recorded *Anopheles* swarm in this specific part of Tanzania, and the first in the country since the last report from north-eastern part of the country in 1983 ^20^. In the initial exploration, several non-mosquito swarms and also culicine mosquito swarms were incorrectly reported by the volunteers as *Anopheles* swarms, but were dismissed by the expert verification teams. The reliability of the volunteers however increased over the course of the surveys.

### *Anopheles* mosquito swarms in the three selected villages

**Figure 3.**
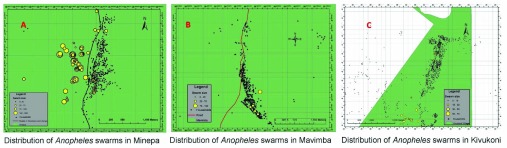
Comparison of swarm distribution between the three study villages (Original figure created by authors of this article).

After the initial exploratory surveys, and confirmation of occurrence of *Anopheles* swarms, subsequent surveys were concentrated in just the three selected villages of Minepa, Mavimba and Kivukoni ( Figure 1). A total of 216 swarms were observed from August 2016 to June 2017. The distribution of mosquito swarms in the study villages is shown ( Figure 3), and was as follows; 58.3% of the swarms (n=126) were found in Minepa village, 11.1% (n=24) were found in Mavimba village and 30.5% (n=66) were found in Kivukoni village. There were more swarms distributed in areas close to the rice fields, at the edges of the villages, compared to areas near human settlements ( Figure 3). All the swarms observed consisted exclusively of *An. gambiae* s.l. We analyzed a sub-sample of 112 *An. gambiae* s.l mosquitoes by PCR to determine actual sibling species, out of which we obtained 97 successful amplifications (86.6%) and 15 non-amplifications (13.4%). All the successful amplifications were determined to be *An. arabiensis* (100%). In one instance in the early days of the surveys, we also collected 13 male *An. funestus* mosquitoes, in an area where we had *An. arabiensis* swarms. Since no repeat observation of *An. funestus*, we are unable to verify presence of swarms of this species here, until further investigations are completed.

### Start times, end times, duration and height of *Anopheles* swarms

**Figure 4.**
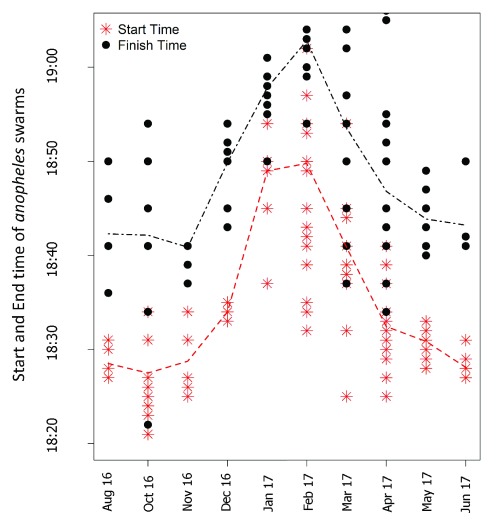
Observed start and end times of *Anopheles arabiensis* swarms in the study villages.

It was observed that swarms began with two to three mosquitoes congregating after sunset, and flying above a swarm marker. Then the numbers increased over the next 10 to 15 minutes, with multiple males and slowly decreased in size then disappeared after 20 (IQR;15-25) minutes. Flying mosquitoes were observed by viewing them against the sunset. Mosquitoes tended to congregate at the mean height (±SD) of 2.74±0.64m from the ground. The swarm size increased and became compact above the marker but they disappeared when it became dark, which was also when swarms observations became impossible. Furthermore, we observed that the start and end time of swarming periods varied across months. Swarms were appearing earlier in the period between August 2016 and January 2017, but later in the period between February and June. Specific start and finish times for swarms during each of the sampling months is shown in Figure 4.

According to weather information archived online by timeanddate.com, the latest time of sunset in the study area (Kilombero Valley, Tanzania) varied in a similar pattern as the start and end times of the observed *Anopheles* swarm sessions. We considered sunset times for all the months of our data collection, starting August, 2016 to June 2017. Between August and November 2016, the latest time sunset time ranged between 18:32Hrs and 18:42Hrs. However, between December 2016 and March 2017, sunset occurred much later, with the latest sunset times between 18:56Hrs and 19:03Hrs. The sunset time was early again between April 2017 and August 2017, when the latest sunset times over the valley ranged from 18:26Hrs and 18:35Hrs: https://www.timeanddate.com/sun/@157407.

### Estimated swarm sizes: data collected through direct visualization by community volunteers compared to data collected using sweep nets

**Figure 5.**
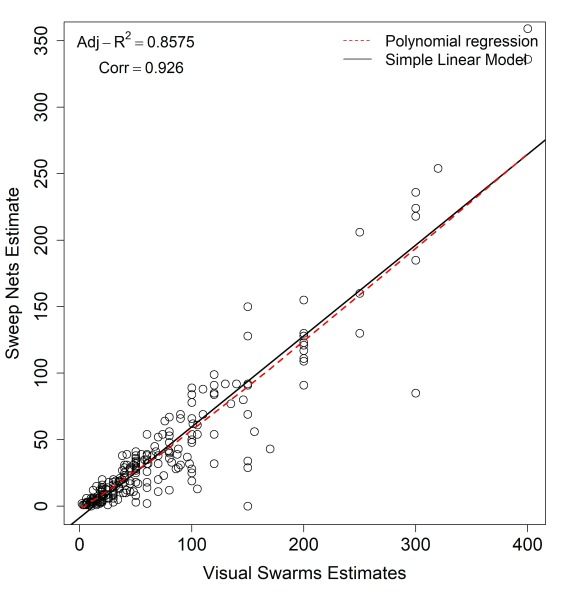
Estimated swarm sizes; and correlation between estimates done by visualization and estimates done using sweep nets.

We estimated swarm sizes from two data sources. First, we used data provided from visual assessments of the volunteers looking at swarms and estimating how many mosquitoes were flying in them at that time. Second, we used data from collections done by the large standardized sweep nets, with which trained volunteers collected the mosquitoes approximately 10 minutes after the start of swarming. Though the values provided from the two data sources were not equal (i.e. visual estimates being consistently higher than sweep net counts), there was a significant linear correlation ( Table 1 and Figure 5) between the visual estimates provided by the community volunteers and estimates obtained from sweep net counts (R ^2 ^= 0.94; P <0.001). There were also variations of swarm sizes observed in different villages, with Minepa village having the largest swarms followed by Mavimba and Kivukoni villages ( Table 1).

**Table 1.** Swarm sizes estimated by either visual observations by trained volunteers, or sweep net sampling in the three study villages (a total of 216 swarms were observed). C.I: Confidence Interval.

	Total number of swarms (% of all swarms observed)	Visual estimates by volunteers (No. mosquitoes/swarm)		Sweep net sampling estimates (No. mosquitoes/swarm)	
		Mean	C.I	Mean	C.I
**Kivukoni**	66 (30.5%)	34.0	31.4 - 36.6	13.1	11.5 - 14.9
**Mavimba**	24 (11%)	38.7	36.3 - 41. 3	20.6	19.9 - 22.5
**Minepa**	126 (58.3%)	95.5	93.8 - 97.3	56.1	54.8 - 57.4

### Results of swarm markers survey

The commonest swarm markers were rice fields (25.6%), burned grounds (17.2%), banana trees (13%), brick piles (8.8%), grass (7.9%) garbage heaps (7.9%), ant hill (7.4%), wood piles (5.6%), demolished houses (1.8%),footpath (1.3%), trenches (1.3%), toilets (1.3) and cowshed (0.5). Table 2 shows the common swarm markers and the frequency of swarms observed in each marker. Most of the swarm locations remained the same, several months after the swarms were first identified and verified.

**Table 2.** Common types swarm markers observed in the three study villages. In brackets, the actual number of swarm markers that had *Anophele* *s* swarms over them.

	Flat contrast swarm markers	Flat no-contrast markers	Elevated contrast markers	Elevated no-contrast markers
**Minepa Village**	Burned ground (17) Well (0) Cowshed (0) Trench (0)	Rice fields (36) Footpath (1) Grass (8)	Bricks (17) Garbage heaps (10) Woodpile (1) Demolished house (2) Ant hill (7) Toilets (3)	Banana trees (24)
**Mavimba Village**	Burned ground (4) Well (0) Cowshed (0) Trench (2)	Rice fields (1) Footpath (1) Grass (5)	Bricks (0) Garbage heaps (4) Woodpile (1) Demolished house (0) Ant hill (6) Toilets (0)	Banana trees (0)
**Kivukoni Village**	Burned ground (16) Well (0) Cowshed (1) Trench (1)	Rice fields (18) Footpath (1) Grass (4)	Bricks (2) Garbage heaps (3) Woodpile (10) Demolished house (2) Ant hill (3) Toilets (1)	Banana trees (4)

### Copulation events, mating status, sexual maturity and parity rates in mosquitoes collected from the swarms

Throughout the study period, we observed and collected a total of 22 copulation events in the swarms. The proportion of female to male *An. arabiensis* mosquitoes in the swarm was 0.004 (28/7114). Of the 28 female *anopheles arabiensis* mosquitoes dissected, 54% (N=15) were determined as nulliparous. The remaining 46% (N=13) were not examinable since the specimen had dried up. We also assessed the male *An. arabiensis* collected from the swarms for sexual maturity. Of these, 95.4% (N=6787) had rotated male genitalia, indicating sexual maturity. We assessed the 28 females recovered from the swarms, to assess whether their spermatheca were already filled with male sperms or not. Of all females dissected, 42.9% (N=12) had evidence of having been inseminated, while the remaining 57.1% (N=16) were not inseminated.

## Discussion

Malaria vector control remains constrained by lack of comprehensive understanding of mosquito ecology, yet such ecological studies are considered a prerequisite for improved control and eventual disease elimination ^7^. Though not widely studied, the mating behavior of malaria mosquitoes may presents some unique opportunities for improved vector control ^13^. In a recent trial in Burkina Faso, it has been demonstrated that targeting *Anopheles* mosquito swarms with effective aerosol insecticide spraying could rapidly crash vector populations in local communities ^14^. Though mating and swarming behaviours of *Anopheles* mosquitoes are among the most important components of the vector reproduction biology ^8^, there have been limited field studies of mosquito swarming in Tanzania, the last published field observations having been in 1983 ^20^. This neglect is mainly because most scientist have not considered swarming behaviour as having any potential opportunities for disease prevention and vector control. On the contrary, studies on mating and swarming behaviours of *Anopheles* mosquitoes in West Africa have already demonstrated that it could possibly be relied upon to control mosquito densities and associated pathogen transmission, by killing mosquito swarms with effective insecticides ^13, 14^.

Our study has yielded evidence of occurrence of the swarms of *An. arabiensis* mosquitoes in the rural south-eastern Tanzania. Furthermore, the study proves that trained volunteers are able to successfully identify and locate swarms in their villages. A map of all swarm locations so far identified has been built-up with the help of the locally recruited volunteers ( Figure 3). Interestingly, most of the swarm locations remained the same, several months after they were first identified. This phenomenon of swarms occurring in the same location is widely reported by other scientists, particularly in west Africa ^26^. In this current study, we characterized the *Anopheles* swarms repeatedly to obtain an initial profile of malaria vector swarms in this area; first evidence of such swarms in the study area.

The commonest swarm markers are shown in Table 2, and were found on landscapes that were not blocked by either trees or houses. Several other studies have also reported the roles of such markers on mosquito swarming ^15^, and here, we relied on similar swarm marker classification as previously used in Burkina Faso ^26^. Mosquitoes preferred to swarm on areas such as rice fields, burned grounds, banana trees, brick piles, garbage heaps and ant hills. It may therefore be possible to predict the distributions of *Anopheles* swarms based on the spatial distribution of the common swarm markers in the village. In this study, once we had an accurate representation of the main swarm markers, it became easier to conduct training programs for the volunteers, who relied heavily on these swarm markers as a guide to search for mosquito swarms in their assigned areas. Second, with a clear definition of swarm markers, it could be possible, as has been demonstrated in some studies to manipulate swarm markers, or create artificial swarm markers to attract *Anopheles* mosquitoes and enhance mating ^16^.

Our study also demonstrated that locally recruited and trained non-entomologist volunteers from the study villages are capable of not only identifying the swarm but also estimating the swarm size and the associated swarm markers in the study village. This idea was derived from an earlier study conducted in the villages where community members were able to identify locations where mosquito densities were high ^22^. In this current study, we initially guided the volunteers on how to identify mosquito swarms and their associated environmental features. This approach confirmed that such community-based volunteers can be relied upon to identify, characterize and map *Anopheles* swarms across villages, so that these swarms can potentially be targeted for control and ensuring its sustainability. An added advantage of this approach is that it also increases local community knowledge on mosquitoes, and the level of community engagement in research increased. By using such participatory approach, we expect that in the time of targeting *Anopheles* swarms e.g. by aerosol spraying, high levels of acceptability could be achieved, and the volunteers could be relied upon to lower implementation costs.

This study did not observe any *An. funestus* swarms. However, 13 male *An. funestus* mosquitoes were collected near the *An. arabiensis* swarms, suggesting possible presence of *An. funestus* swarms in the area. Unfortunately, this was only one instance and could not be verified during this study. It may be possible that this was a separate distinct *An. funestus* swarm that was formed close to *An. arabiensis*. Since they were all male *An. funestus*, we speculate that *An. funestus* swarms also occur in the area, and that extended surveys could reveal greater details of their swarming behaviours. It is therefore recommended that more detailed surveys should be conducted to understand the swarming behaviours of *An. funestus* in the area. Evidence by other studies in Africa suggest that swarms of *An. funestus* appear in a similar way to those of *An. gambiae* ^21^. Since it is know that *An. funestus* contributes to the significant amount of the remaining malaria transmission in this area ^23^, it is therefore suggested that any control measures targeting *An. gambiae* without affecting *An. funestus* will have little impact on the ongoing malaria transmission in this area. More attention should be put on how to identify swarms of *An. funestus* and their associated environmental features.

Consequently presence of dragonflies, though not initially targeted for observation, was an indication of swarm presence in many locations, though in this case we have not considered it as a physical swarm marker. Similar observations were also reported by Sawadogo *et al*., ^15^ in their studies in Burkina Faso. Furthermore, human movements, sounds, strong wind and rainfall were found to affect mosquito swarming behaviours. For instance, in some cases when swarm sampling was conducted during the rainfall, we rarely observed swarms even in the previously confirmed swarms. Dragon flies and other predators could thus be considered good indicators of presence of mosquito swarms in certain areas. Lastly, in this study, most of the swarms were found and confined at the edge of the village and in the rice fields, this could mostly be attributed to the fact that swarms prefers to the location that are not disturbed by human activities such as movements.

Overall, the swarming behavior of *An. arabiensis* in the valley is now clearly tractable. Further investigations should explore opportunities on whether new interventions exploiting this phenomenon, such as targeted aerosol spraying of swarms, could significantly disrupt malaria transmission.

## Conclusions

This is the first report of *Anopheles* swarms in Tanzania, in more than three decades. The study provides evidence that the swarms can be identified, characterized and quantified by trained community-based volunteers; and opens potential new opportunities for targeting male malaria mosquitoes to improve disease control. Further investigations are recommended to further characterize the swarms in the area and to verify whether other residual malaria vector populations such as *An. funestus* also form similar mating swarms. More importantly, future studies should seek to demonstrate whether new interventions exploiting this phenomenon, such as targeted aerosol spraying of swarms, could significantly disrupt malaria transmission.

## Data availability

The data referenced by this article are under copyright with the following copyright statement: Copyright: © 2017 Kaindoa EW et al.

The data used in this manuscript have been uploaded online to the Ifakara Health Institute, DOI, 10.17890/ihi.2017.06.99 ^34^, under a CC0 1.0 license.

The terms and conditions that users need to agree to in order to download the data, can be viewed here.
