# Supplementary material for: New evidence of mating swarms of the malaria vector, Anopheles arabiensis in Tanzania
Source: Wellcome Open Res. 2017 Sep 22;2:88. [Version 1] doi: 10.12688/wellcomeopenres.12458.1 (PMC5691375; doi:10.12688/wellcomeopenres.12458.1)
Supplement: Peer review history

# Peer review: Referee response for version 1

**DOI:** 10.21956/wellcomeopenres.13490.r27476

**Published:** 2017-11-15

**Authors:** Jacques Derek Charlwood

**Affiliations:** 1 Institute of Hygiene and Tropical Medicine (IHMT), Global Health and Tropical Medicine (GHTM), Nova University of Lisbon, Lisbon, Portugal

This paper represents a lot of work. The problem with studying swarming behavior (as opposed to mating behavior) is the difficulty of having sensible questions to ask.  I have watched and filmed swarms of anophelines for many hours (mainly in Mozambique) and still am not sure what the most relevant ones, at least without sophisticated equipment, might be. There is always more perspiration than inspiration. This paper describes the basics of swarms of *Anopheles arabiensis* but I have a number of questions regarding some of the information in the paper. For example, having recently observed swarms of *An. arabiensis* in Eritrea (but only over a period of two weeks and in a single village) I remain at a loss regarding definitions of swarm markers for this species. The swarms I observed occurred over a very slightly darker area of sand in a dry riverbed but the mosquitoes did not alter their behavior when either a light cloth or dark T-shirt was placed under the swarming mosquitoes.  I am therefore of the opinion that I have no real clue what the insects were using to maintain station. (This is unlike the situation with *An. coluzzii, *which will follow a dark cloth or *An. pharoensis *which follow light ones). I am, therefore, not convinced that the so-called markers described in the present study were actually being used by the *An. arabiensis. *Their wide variety indicates that they were just in the area and that something else was the actual ‘marker’.  Only by manipulating apparent markers and seeing their effect on the swarms can we be sure that they are for real.

 In Eritrea males left their diurnal resting sites once light levels had fallen to around 520 Lux (which occurred at different times according to whether it was cloudy or not) and immediately started swarming. There was no delay.  As the authors indicate swarming did not last long but at the same time, as they also point out, the cessation of swarming was coincident with difficulty of actually observing the insects so it is difficult to be sure that activity had ceased. Swarms of *An. funestus, *monitored with a video camera with infra-red capabilities in Mozambique, continued beyond the time that they could be seen with the naked eye. So a caveat regarding ending time should be included. The change in start times according to sunset times was also recorded in *An. coluzzii* from São Tomé and should perhaps be referenced.

 Sweep netting, of course, does not remove all of the insects from a swarm. A better alternative is to take a flash photograph and then use a program like imageJ to count objects of different sizes in the image (which can be compared to counts done by hand). Since most pictures are taken from below the swarm the only objects in such pictures are the mosquitoes. Should two insects overlap then their combined size is larger than individual insects and so they are considered by the program to be a separate category. It may even be possible to use the camera on a smartphone, now widely available, to take such pictures.

 Similarly I saw (perhaps) just a single pair in copula in Eritrea. These are normally very easy to see since the pair is larger and flies more slowly than individual insects (the male and female flying in different directions). They almost invariably leave the swarm (the female dragging the male behind her. It is only perhaps when a small female mates with a larger male that they continue in the swarm, the male in this case pulling the female behind him). In all their observations the authors report ‘observing and collecting 22 copulation events’. (Difficult to know how you collect an event). I think that this begs the question as to whether, like ‘love and marriage’ these phenomena really go together (and the ones in Eritrea) really were ‘mating swarms’.  In São Tomé pairs dropped out of the swarms ‘like flies’ (if you’ll excuse the pun).  Something more is going on – but we don’t know what it is. I think it is a misnomer to call them mating swarms. Indeed it is worth discussing the fact that so few mating pairs were seen. It is possible that mating was largely taking place in swarms elsewhere.

 To watch swarms and note their behavior does indeed not require that much professional input. In São Tomé the swarm with mating was discovered by a two-year-old child who had to really persist to get the ‘professionals’ attention!

 The authors also found that 12 of 28 females collected from swarms had been inseminated. Like one of the other reviewers I also find this very strange. Did these insects have mating plugs? What were they doing there?  Elsewhere, as mentioned above, pairs in copula leave the swarm before the male ejaculates. The females do not return (as shown by the absence of multiple mating plugs in the many hundreds of females that have been examined).  My own suspicion would be that the volunteers were being bitten and included these females in the sample.

 It is also interesting that males with un-rotated terminalia were collected. Since these males would probably have ecloded the previous evening they would have been almost 24hrs old at the time of the swarm it implies that rotation takes longer than a day. But I was of the impression that that was all the time that was needed. Some comment on this might also be informative.

 Mosquitoes swarm close to where they rest. Rather than attempting to target swarms per se a better and more sustainable method of control would be to find the resting site (and here the swarming site could be used as an indicator of nearby resting sites) and intervene there. If, as is likely, there are common characteristics between resting sites (a dark background perhaps) then it might also be possible to develop and deploy artificial resting sites that might be treated with a non-repellent insecticide or a factor that the males might transfer to the females that they mate with to affect them. Since newly emerged females also often share the same resting site as the males such an intervention would work against them too.

 I agree with the comments of the other referees in that the article could be a lot shorter, that the figures could be improved and the text be made more concise.

I have read this submission. I believe that I have an appropriate level of expertise to confirm that it is of an acceptable scientific standard, however I have significant reservations, as outlined above.

# Peer review: Referee response for version 1

**DOI:** 10.21956/wellcomeopenres.13490.r26301

**Published:** 2017-10-20

**Authors:** Tovi Lehmann

**Affiliations:** 1 Laboratory of Malaria and Vector Research, National Institute of Allergy and Infectious Diseases (NIAID), National Institutes of Health (NIH), Rockville, MD, USA

This is a valuable description of A. arabiensis mating swarms in Tanzania based on one year swarm survey in 3 villages. The motivation of the study, as stated by the authors is that after the pioneering work of Marchand and later of Charlwood in East Africa, there have been no studies of swarming behavior of African anophelines in that region. The absence of observations on swarms of A. gambiae s.l., despite some efforts, led some to doubt the relevance of swarms to mating in these species, especially in that region. By replicating older results, the authors confirmed the role of swarming behavior to mating across Africa. Thus, the results, mostly replicate previous studies including: swarm formation time after sunset, swarm size range, and association with ground markers. The study relied on trained local village observers/collectors following earlier studies in Mali and Burkina Faso (cited). The paper reads easily (and pleasantly) but it could be improved by a substantial reduction in length, adhering to concise and more precise scientific writing, and possibly addressing specific questions/hypotheses, as suggested below.

 Major comments:

1. Because this study is a descriptive survey of swarms that mostly replicate previous studies (it could cite other studies for details on the Methods), it would better fit a “Research Note” or a much shorter article. Suggestions to accomplish that include replacing Fig. 1 with a brief text and removal of Fig. 2, which is not informative. The Methods section could be reduced to 1/4 of its current length. The detailed description of the training for swarm spotters/collectors, should be summarized in 3 sentences including the description of training in standard entomological and field ecology methods, which would better rely on citations. The first section of the Results “Results of exploratory surveys: initial evidence of Anopheles swarms,” (which repeats information given in the Methods) could be removed (also from the Methods section) or replaced by a single sentence such as: “The initial exploration, confirmed that Anopheles swarms do occur in the study area.” Additionally, there are repetitions and unnecessary details that dilute the main message.
  Certain terms must be defined accurately. For example a “swarm” may represent a single observation of males flying stationary above a marker in a single evening or to the site, where such aggregations were observed on multiple evenings. When stated that 216 swarms were observed, it is not clear which definition was used. Likewise, the term “sub-village” is vague. Please define its area.
  Despite the long Method section, I could not find a summary of the sampling effort i.e. the number of swarm collectors operating throughout the year in every village, which is essential. Did the same team passed between villages in different times or they were divided between the villages? How were the swarm spotter/collectors been evaluated in terms of quality and efficiency of work?
2. Interestingly, large differences were found among villages in the number of swarms and their sizes. Could these differences reflect different density of A. arabiensis overall in these villages (it would be helpful to include average indoor density if such data exist)? Were the sampling effort the same between villages in terms of the number of collectors/observers, duration of activity, and their work-quality, or hospitality of local residents? Were the sampling conducted at the same time across all villages? For example, if the survey in one village occurred in a dryer period, it might account for the lower number and sizes of the swarms. What other factors might explain that variation (e.g., availability of suitable markers)?

Minor/Specific points:

 Background

- “Most insects mate in swarms, whereby dispersed populations aggregate at specific times...” – this statement might meant to refer to diptera or to culicids. I doubt it holds for all insects. The references given focused on dipterans. Please check and confirm.
- “Targeting swarms to deplete mosquito densities indeed offer unrivalled opportunities to drastically reduce mosquito-borne pathogen transmission” – in what way it is unrivalled?
- “Such an approach has proven effective against some Anopheles mosquitoes on a limited scale in Burkina Faso14, but needs to be validated for other vector species in other areas. ..thus targeting them could be easily achieved by trained community volunteers. … easy control target.” I afraid the scalability of this approach for regional, country and continental control is a heavy challenge that cannot be described as “easy”. There are other control strategies that can be effective in a single village or few villages, but can’t be effectively scaled up. For example draining or insecticide-treating all larval sites in certain villages to drastically reduce transmission, but unless the scalability issue has been addressed, the promise if this strategy is doubtful. Therefore the promise of the proposed new approach should be considered seriously with its limitations.
- “ ..a combination of crowd-sourced community knowledge22, intensive field surveys and expert advice” –  specifically what “crowd sourced community knowledge” was obtained.

Results:

- Figure 3 – the legend is very small and nearly impossible to read. It would help to have on large-font and uniform legend for all 3 villages. Please clarify if (i) all houses in the area depicted are shown, ie., there are no other houses (not shown) regardless if they “officially belong” to the village, gpsed etc.. Also, what is the reason that the background to C is different? The landscape around the villages may be better conveyed using Google Earth (than a green color). Figure 3 summarizes large body of spatial information. It might be interesting to extract measures of inter-swarm distances, its seasonal variation, and effect of swarm size, as well as the variation between villages.
- Figure 4. The authors explain the variation in the swarming time by the change in sunset time. Thus, please add the corresponding line (curve) showing the sunset time and include the correlation between these variables in the Figure.
- Figure 5. Unless many observations are on top each other, the number of swarms observed appear to be 4-6/month, which amount to mere ~60 observations total rather than 216 as understood from the top of the results. If there are on top of each other, the author can consider jittering observations and changing their symbol to better appreciate the weight of each value. Please clarify.
- Table 2 shows variation in swarm occurrence on different markers. I suggest that authors reshape the table by listing all the swarm markers along the rows (in groups based on their classification) and have the columns show the percent (and total number) of swarms above each marker across villages. This will allow the reader to compare the ranking of the markers. Moreover, it might be helpful to know how many such markers exist in the surveyed area. For example if there are no rice fields or banana trees in one village and many in another, it will be difficult for the mosquitoes to express their preference for banana tees, etc.  The interpretation of this table can be expanded in the discussion.
- The authors may want to comment on whether the collection of a proportion of inseminated females in swarms as well as some funestus specimens might reflect collection of mosquitoes that are not related to the swarming activity.

I have read this submission. I believe that I have an appropriate level of expertise to confirm that it is of an acceptable scientific standard, however I have significant reservations, as outlined above.

# Peer review: Referee response for version 1

**DOI:** 10.21956/wellcomeopenres.13490.r26445

**Published:** 2017-09-28

**Authors:** Edward D Walker

**Affiliations:** 1 Michigan State University, Department of Microbiology and Molecular Genetics, East Lansing, MI, USA

This quite interesting and pleasantly written article summarizes a useful study combining observational science, citizen science, community engagement, Anopheles mosquito swarming and mating behaviour, and empirical analysis.  The study is mensurative, that is, depending upon various methods of various degrees of sensitivity in measuring and recording observations and events.  The distribution of locations of mating swarms of male Anopheles arabiensis is presented quite well and reliably.  One problem is the issue of recorder bias; how likely are the observers to go to (say) sites most convenient for them to visit as opposed to sites difficult to visit.  But this problem seems to have been surmounted here and the authors are not unaware of it.  The observations on swarm composition (e.g., sex ratio), timing of formation and deformation, and density (number of mosquitoes comprising them) are all useful.  This study might lead to new tools for vector control based upon these observations and related data.

 Conclusions from data are again based on observational inferences but this kind of study is useful precisely for that reason.

 Certain matters remain unclear and these have to do in particular with the location of swarms and what is meant by swarm markers.  For example, a category of swarm marker is rice field and another is banana tree and another is toilet by which we must mean latrine shed.  These are all entirely different environmental features with very different shapes, sizes, compositions, origins, and so on.  From the observations one cannot make a strong conclusion about the particular configuration of what is meant by "swarm maker" typologically.  That is, are swarm markers typically of a certain configuration or do swarms form just about anywhere the initial males starting them begin to fly at a particular spot?  The authors should clarify this issue as a point of discussion.  Secondly is the problem of what is meant by contrast or no contrast swarm markers.  I do not know what this means.  Do they mean contrast with a horizon, contrast between two hues or shades, ... ?  As a a means of providing a particular attribute of a typical swarm marker, the use of this descriptor of contrast is vague.

 The writing style is generally quite good and the manuscript needs little adjustment for editing.  The caption legends for Figure 5 are awkward.  The X axis could simply read Visual Estimate and the Y axis could read Sweep Net Estimate.  I see no reason to include a polynomial fit to the data on this figure because the relationship looks plainly linear.  There seem to be two data points tucked up high in the upper right corner but they are obscured by the regression information.

 In the last paragraph of the Results section, the following sentence should be revised: "Of the 28 female anopheles arabiensis mosquitoes dissected, 54% (N=15) were determined as nulliparous. The remaining 46% (N=13) were not examinable since the specimen had dried up."  It should read as follows: "of the 28 female An. arabiensis dissected, fifteen were nulliparous and the remainder were too dry to determine parity status."

 In Table 2, the structure of the table needs some changes reflecting the clarification of the contrast and no contrast comment above.  By cowshed must be meant the area of land where cows are stanchioned at night.  This is not a 'cowshed' in the usual sense but rather a flat piece of ground where the animals are kept at night.  If it is a kraal type of situation, there may be some living or nonliving fence in place in which case it cannot be flat.  Otherwise, explain how a cowshed is flat.  Under elevated contrast markers, I would like to know if by ant hills is meant termite mounds.  Please clarify.  My experience is east Africa is that ant hills are quite low to the ground and indiscernible whereas termite mounds are of course tower like structures and clearly elevated.

 Some banana trees are quite tall.  How did the citizens with the sweep nets manage to sample over them?

 It is interesting that Figure 3 shows that in some locations the swarms form independently or distance from houses whereas in other locations the swarms form mostly amongst dense clusters of houses.  Compare panel 1 and panel 2, for example.  Why is this?

 Thank you for giving me this opportunity to review this interesting and useful manuscript.

I have read this submission. I believe that I have an appropriate level of expertise to confirm that it is of an acceptable scientific standard.
